# Engineered Nanobody-Bearing Extracellular Vesicles Enable Precision Trop2 Knockdown in Resistant Breast Cancer

**DOI:** 10.3390/pharmaceutics17101318

**Published:** 2025-10-11

**Authors:** Jassy Mary S. Lazarte, Mounika Aare, Sandeep Chary Padakanti, Arvind Bagde, Aakash Nathani, Zachary Meeks, Li Sun, Yan Li, Mandip Singh

**Affiliations:** 1 Institute of Public Health, College of Pharmacy and Pharmaceutical Sciences, Florida A&M University, Tallahassee, FL 32307, USA; jassy.lazarte@famu.edu (J.M.S.L.); mounika1.aare@famu.edu (M.A.); sandeep.padakanti@famu.edu (S.C.P.); arvind.bagde@famu.edu (A.B.); akash.nathani96@gmail.com (A.N.); zachary5.meeks@famu.edu (Z.M.); 2 Department of Chemical and Biomedical Engineering, FAMU-FSU College of Engineering, Florida State University, Tallahassee, FL 32310, USA; li.sun@med.fsu.edu (L.S.); yli@eng.famu.fsu.edu (Y.L.); 3 Department of Biomedical Sciences, College of Medicine, Florida State University, Tallahassee, FL 32310, USA

**Keywords:** Trop2, nanobody, extracellular vesicles, letrozole-resistant breast cancer, target therapy

## Abstract

**Background/Objectives**: Trophoblast cell surface antigen 2 (Trop2), a transmembrane glycoprotein overexpressed in a broad spectrum of epithelial malignancies but minimally expressed in normal tissues, has emerged as a clinically relevant prognostic biomarker and therapeutic target, particularly in breast cancer. This study aims to develop an enhanced way of targeting Trop2 expression in tumors and blocking it using extracellular vesicles (EVs) bioengineered to express a nanobody sequence against Trop2 (NB60 E). **Methods**: Here, a plasmid construct was designed to express the Trop2 sequence, NB60, flanked with HA tag and myc epitope and a PDGFR transmembrane domain in the C-terminal region, and was transfected into HEK293T cells for EVs isolation. The potency of NB60 E to knock down Trop2 in letrozole-resistant breast cancer cells (LTLT-Ca and MDA-MB-468 cells) was initially investigated. Thereafter, the effects of NB60 E on the cell viability and downstream signaling pathway of Trop2 via MTT assay and Western blotting were determined. Lastly, we also examined whether NB60 E treatment in Jurkat T cells affects IL-6, TNF-α, and IL-2 cytokine production by enzyme-linked immunosorbent assay (ELISA). **Results**: Results revealed treatment with NB60 E significantly reduced surface Trop2 expression across both cell lines by 23.5 ± 1.5% in MDA-MB-468, and 61.5 ± 1.5% in LTLT-Ca, relative to the HEK293T-derived control EVs (HEK293T E). NB60 E treatment resulted in a marked reduction in LTLT-Ca cell viability by 52.8 ± 0.9% at 48 h post-treatment. This was accompanied by downregulation of key oncogenic signaling molecules: phosphorylated ERK1/2 (p-ERK 1/2) decreased by 30 ± 4%, cyclin D1 by 67 ± 11%, phosphorylated STAT3 (p-STAT3) by 71.8 ± 1.6%, and vimentin by 40.8 ± 1.4%. ELISA analysis revealed significant decreases in IL-6 (−57.5 ± 1.5%, 7.4 ± 0.35 pg/mL) and TNF-α (−32.1 ± 0.3%, 6.1 ± 1.2 pg/mL) levels, coordinated by an increase in IL-2 secretion (22.1 ± 2.7%, 49.2 ± 1.1 pg/mL). Quantitative analysis showed marked reductions in the number of nodes (−45 ± 4.4%), junctions (−55 ± 3.5%), and branch points (−38 ± 1.2%), indicating suppression of angiogenic capacity. In vivo experiment using near-infrared Cy7 imaging demonstrated rapid and tumor-selective accumulation of NB60 E within 4 h post-administration, followed by efficient systemic clearance by 24 h. The in vivo results demonstrate the effectiveness of NB60 E in targeting Trop2-enriched tumors while being efficiently cleared from the system, thus minimizing off-target interactions with normal cells. Lastly, Trop2 expression in LTLT-Ca tumor xenografts revealed a significant reduction of 41.0 ± 4% following NB60 E treatment, confirming efficient targeted delivery. **Conclusions**: We present a first-in-field NB60 E-grafted EV therapy that precisely homes to Trop2-enriched breast cancers, silences multiple growth-and-invasion pathways, blocks angiogenesis, and rewires cytokine crosstalk, achieving potent antitumor effects with self-clearing, biomimetic carriers. Our results here show promising potential for the use of NB60 E as anti-cancer agents, not only for letrozole-resistant breast cancer but also for other Trop2-expressing cancers.

## 1. Introduction

Chemotherapy, radiotherapy, hormonal therapy, and surgery remain the standard paradigm for antitumor therapeutics. Although these standard treatments have unquestionably improved the survival of many breast cancer patients, their effectiveness diminishes as tumors adapt and develop drug resistance, creating an ongoing demand for alternative strategies capable of restoring durable disease control [1,2,3]. The emergence of new strategies for tumor-targeting paved the way for developing novel targeted therapeutics. Targeted therapeutics develop by first identifying specific moieties that are highly (if not exclusively) expressed by the tumor and are strongly associated with functionalities involving tumor growth, proliferation, or metastasis [4]. A prototypical example of such a marker is trophoblast cell surface antigen 2 (Trop2), a 36 kDa single-pass transmembrane glycoprotein first described nearly forty years ago on placental trophoblasts and later re-identified under multiple aliases, including tumor-associated calcium signal transducer 2 (TACSTD2), GA733-1, epithelial glycoprotein-1 (EGP-1), and membrane component chromosome 1 surface marker 1 (M1S1) [5,6]. Subsequent studies confirmed a developmental role for Trop2 during embryogenesis [7], yet the protein’s best-documented function is its pervasive overexpression in a broad spectrum of solid epithelial malignancies including oral, head-and-neck, thyroid, lung, esophageal, gastric, colorectal, pancreatic, breast, renal, uterine, cervical, and ovarian carcinomas, as well as gliomas and several sarcoma subtypes [8,9,10]. Expression of Trop2 in normal tissues signals cells for self-renewal, proliferation, invasion, and survival [11,12]. When overexpressed, however, Trop2 activates oncogenic signaling that accelerates the cell cycle, enhances migration and invasion, drives epithelial-to-mesenchymal transition, and confers resistance to chemo- and radiotherapy [13]. In breast cancer, Trop2 is directly linked to various facets of cancer progression, from promoting cell proliferation to resisting therapies [5]. Moreover, elevated Trop2 is associated with unfavorable prognostic markers that include larger tumor size and increased risk of recurrence [9,14,15]. Previous studies have detailed the recruitment of multiple signaling pathways that explain the mechanism behind Trop2-mediated tumor growth, proliferation, and metastasis [16,17,18]. These functional mechanisms of Trop2 on tumor progression make it a promising therapeutic target. Furthermore, while normal human tissues exhibit limited expression of Trop2 [19], in breast cancer, Trop2 expression was observed to be more pronounced [20]; thus, the use of Trop2 presents a potential targeting module for diagnosis or tumor therapy.

Therapeutic platforms directed against Trop2 have centered primarily on monoclonal antibodies (mAbs) and related formats. Notably, T-cell-redirecting bispecific antibodies engaging both Trop2 and CD3 suppress tumor growth in triple-negative breast-cancer (TNBC) lines and primary explants [21]. The most successful case of Trop2-targeted therapy is Sacituzumab Govitecan (SG; Trodelvy or IMMU-132), which has been approved by the U.S. Food and Drug Administration (FDA). This ADC, formulated for treating patients with metastatic TNBC, consists of a humanized anti-Trop2 monoclonal antibody (mAb) and the topoisomerase I inhibitor drug SN-38 [22]. Other promising ADCs are under investigation such as Datopotamab deruxtecan (Dato-DXd or DS-1062a) and SKB264 (AKB264) [23,24], both of which explicitly target Trop2-expressing cancer cells to ensure precise delivery of the drug payload to the tumor. The ADC format mainly relies on the preparation of the Trop2-specific mAbs. Setbacks regarding this primarily stem from the intrinsic properties of mAbs, which include limited tumor penetration partially due to their large size [25] and the high cost for production; their in vitro production requires sophisticated eukaryotic machinery, which is why several strategies to reduce production cost have been developed, just like the case of Rituximab [26]. The antibody portion of all current Trop2-directed ADCs are IgG-type mAbs, which have a full length of 150 kDa; this size prevents the penetration of mAbs into solid tumor tissues. One strategy for dealing with this problem is by reducing the molecular size. Nanobodies (Nbs), or VHHs, derived from camelid heavy-chain-only antibodies, weigh ~15 kDa yet retain full antigen affinity and specificity [27,28]. Nanobodies also have high solubility and inherent domain stability under extreme conditions, which ensures binding activity in the tumor microenvironment. Their exceptional solubility, stability, and ease of microbial expression yield pharmacokinetic and economic advantages, enabling deeper tumor diffusion while lowering production costs [29]. Pairing Trop2-specific nanobodies with an efficient delivery vehicle such as EVs may amplify Trop2 blockade and enhance receptor clustering and internalization. Although EVs are larger than monoclonal antibodies, the use of nanobodies as targeting moieties is advantageous because their small size and single-domain structure enable efficient genetic fusion, stable display, and higher packing density on EV membranes, overcoming the technical limitations associated with full-length antibodies.

Extracellular vesicles (EVs) or EVs, endogenous lipid-bilayer vesicles 40–160 nm in diameter, have emerged as versatile nanocarriers able to transport small-molecule drugs, proteins, and nucleic acids [30]. Recently, EVs have been investigated as drug delivery vehicles that can deliver payloads (i.e., chemotherapy drugs and biologics) in lung cancer models [31,32,33]. Their small size, good biocompatibility, low toxicity, low immunogenicity, and an unrivaled ability to cross biological barriers such as the blood–brain barrier and stromal matrices suggest their potency as drug carriers for cancer therapy [34,35]. Since EVs derive their cargo materials, such as cell-specific proteins and genetic materials, from the parental cells, surface modification by bioengineering can be performed to modify the secreted EVs. These favorable characteristics of the EVs and Nbs strengthen our hypothesis that EVs can be bioengineered to express Trop2-specific nanobodies, allowing for a more targeted antitumor therapy for breast cancer.

While nanobodies alone indeed exhibit deep tumor penetration and high antigen specificity, they suffer from rapid systemic clearance and short circulation half-life. NB60-targeted EVs can potentially enhance receptor clustering and internalization. This platform is modular and can be further expanded for cargo loading or dual-targeting strategies, offering advantages over naked nanobody therapy alone and other existing Trop2-targeted therapies. Furthermore, NB60-targeted EVs can be further improved by bioengineering them to express immune stimulants, in addition to carrying therapeutic agents such as chemotherapeutics, siRNAs, and miRNAs that target specific cancer markers. This enhancement addresses a limitation of current Trop2-targeted therapies, which lack these additional features. Currently, Trop2-targeted therapy on the market utilizes Trodelvy^®^, an antibody–drug conjugate. Its therapeutic outcome is limited because each antibody can carry only a finite amount of cytotoxic payload, which constrains efficacy and can contribute to systemic toxicity.

Letrozole-resistant MCF7 cell lines, such as LTLT-Ca, have been developed to investigate the mechanisms underlying resistance to aromatase inhibitors. These cells are derived from the estrogen receptor-positive MCF7 breast cancer cell line and have acquired resistance through prolonged exposure to letrozole. Notably, LTLT-Ca cells retain estrogen receptor positivity, but exhibit altered signaling pathways that confer resistance. Studies have shown that these cells display increased activation of the PI3K/Akt pathway, contributing to their survival and proliferation despite estrogen deprivation [36,37]. Treatment-resistant cells, such as LTLT-Ca, have developed the ability to migrate and invade surrounding tissues more rapidly, which increases cancer aggressiveness and the potential for metastasis. This concerning characteristic prompted us to use these cells as a model for this study. Our goal was to demonstrate the efficacy of NB60 E in reversing resistance and promoting the regression of cancer cells in this breast cancer cell line.

In this study, we developed EVs bioengineered to display a well-characterized Trop2-specific nanobody sequence (NB60 E). Following large-scale harvest and ultracentrifugation, NB60 E were characterized for size distribution and ζ-potential. We assessed Trop2 knockdown by Western blot and examined downstream effects on viability, apoptosis, and oncogenic signaling in LTLT-Ca cells. In parallel, we profiled cytokine release in Jurkat T cells to gauge immune modulation and conducted HUVEC tube-formation assays to probe anti-angiogenic potential.

## 2. Materials and Methods

### 2.1. Materials

HEK293T (CRL-3216), MDA-MB-468 (HTB-132), and Jurkat T (TIB-152) cells were purchased from American Type Culture Collection (Manassas, VA, USA). HUVEC-Umbilical Vein Endothelial Cells-EGM-2 (C2517A) were purchased from Lonza (Walkerville, MD, USA). LTLT-Ca cells were obtained from the lab of Dr. Syreeta L. Tilghman and the cells were prepared based on the paper by Jelovac et al., 2005 [37]. Primary antibodies specific to proteins: myc (Cat # 2276), Trop2 (Cat # 90540), Alix, (Cat # 92880), TSG101 (Cat # 72312), CD81 (Cat # 56039), CD9 (Cat #13403), Calnexin (Cat # 2679), Vimentin (Cat # 5741), total ERK 1/2 (Cat # 4695), phosphorylated ERK 1/2 (p-ERK1/2, Cat # 9101), cyclin D1 (Cat # 55506), Caspase 3 (Cat # 9662), IL-6 (Cat # 12912), Stat3 (Cat # 12640), phosphorylated Stat3 (Cat # 9145), TGF-β (Cat # 3711), NF-κB (Cat # 8242), phosphorylated NF-κB (Cat # 3033), secondary antibodies: anti-mouse IgG, HRP-linked Antibody (Cat # 7076), anti-rabbit IgG, HRP-linked Antibody (Cat # 7074) and housekeeping protein, β-actin (Cat # 4970) were purchased from Cell Signaling Technology (Danvers, MA, USA).

### 2.2. Cell Culture

HEK293T and MDA-MB-468 cells were cultured in high glucose Modified Eagle Medium (DMEM) (Genesee Scientific, San Diego, CA, USA) supplemented with 10% heat-inactivated fetal bovine serum (FBS), 100 U/mL penicillin, and 100 μg/mL streptomycin (Genesee Scientific, San Diego, CA, USA). Jurkat T cells were grown in RPMI-1640 Medium supplemented with 10% heat-inactivated fetal bovine serum (FBS), 100 U/mL penicillin, and 100 μg/mL streptomycin (Genesee Scientific, San Diego, CA, USA). To maintain the letrozole resistance of the LTLT-Ca cells, they were cultured in MEM (Richter’s modification) (Cat. # A1048801, Life Technologies, Carlsbad, CA, USA) supplemented with 10% charcoal-stripped heat-inactivated fetal bovine serum (CS-FBS), 100 U/mL penicillin and 100 μg/mL streptomycin, 750 µg/mL Geneticin^TM^ Selective Antibiotic (G418 sulfate) (Cat. # 10131027, Life Technologies, Carlsbad, CA, USA), and 1 µM letrozole (Cat. # L6545-50MG, Sigma-Aldrich, St. Louis, MO, USA). The cells were grown at 37 °C in 5% CO_2_/95% humidified air. HUVECs were maintained in EBM-2 Basal Medium (CC-3156) supplemented with EGM-2 SingleQuots Kit Growth Factors (CC-4176) (Lonza, Walkersville, MD, USA).

### 2.3. Establishment of Cell Line Expressing Trop2 Nanobody (NB60)

**Figure 1:**
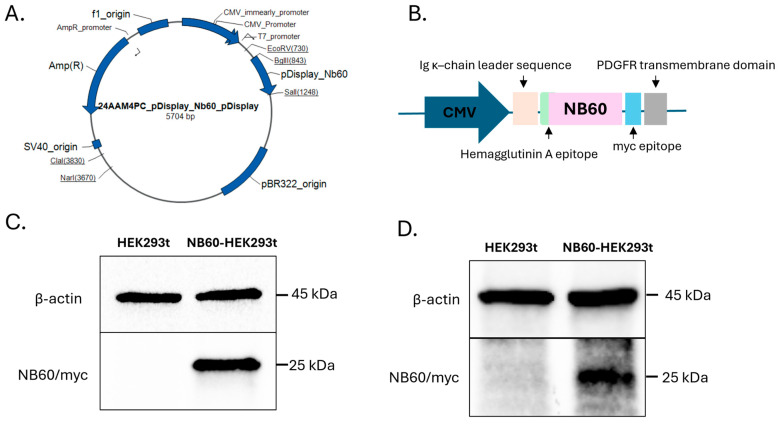
Establishment of NB60-expressing HEK293T cell line. (**A**) The vector map of the plasmid construct, pNB60. (**B**) The specific components of the plasmid construct essential for the expression of Trop2 (NB60) on the surface of the EVs. (**C**) Expression of NB60 in HEK293T cells after transfection. (**D**) Expression of NB60 in HEK293T cells after geneticin (200 μg/mL) selection for 30 days. Legend: HEK293t—un-transfected HEK293T cells, NB60-HEK293t—pNB60-transfected HEK293t cells.

The plasmid construct, pDisplay NB60 (pNB60) with HA tag and myc epitope, was purchased from ThermoFisher Scientific (Carlsbad, CA, USA). The NB60 sequence was derived from the published paper by Hu et al., 2022 [38]. The pNB60 is designed with several important features including (a) Ig κ-chain leader that directs the protein to be secreted extracellularly after transfection, (b) hemagglutinin A epitope for protein detection, (c) Trop2 nanobody sequence, NB60, (d) myc epitope for protein detection, and (e) PDGFR transmembrane domain that helps anchor the protein to the cell membrane (Figure 1B). The pNB60 was transfected into human embryonic kidney (HEK) 293t cells in a 24-well plate using Lipofectamine™ 3000 Transfection Reagent (Invitrogen, Life Technologies, Carlsbad, CA, USA). The DNA–lipofectamine complex was replaced with DMEM (2% FBS) after 4 h of incubation. Two days post-transfection, the cells were collected, and protein samples were prepared for Western blotting experiments. Once the expression of NB60 was verified, the transfected cells were subjected to geneticin (200 μg/mL) selection for 30 days to ensure NB60-expressing cell line. To further verify NB60 expression after antibiotic selection, another round of Western blotting was performed. NB60-HEK293T cells denote pNB60-transfected cells, while HEK293T signify HEK293T cells that were not transfected and were used as negative control.

### 2.4. NB60-HEK293T Cell Culture in 500 mL PBS-Vertical Wheel (VW) Bioreactor

Geneticin-selected NB60-HEK293T cells were grown in tissue culture flasks using DMEM media containing 2% EVs-free FBS, 1% penicillin-streptomycin, and 200 μg/mL geneticin, and incubated at 37 °C in 5% CO_2_/95% humidified air and 20% O_2_. A total of 10 × 10^6^ cells or 20,000 cells/mL were inoculated into the PBS-VW bioreactor. The agitation speed was set to 25 rpm (0.12 dyn/cm^2^) for 14 days. Samples, 500 μL of media, were collected every four days from PBS-VW bioreactor to obtain an understanding of the metabolism of the cells and to identify the number of days after which the media should be changed. The levels of glucose, lactate, glutamine, and ammonium as well as electrolytes including sodium, potassium, and calcium were evaluated for the collected media using BioProfile Flex2 analyzer (Nova Biomedical, Waltham, MA, USA).

### 2.5. Collection of EVs from Cell Culture Supernatants

Collection of EVs was performed by growing 10 × 10^6^ cells of HEK293T and NB60-HEK293T cells in a 500 mL bioreactor vessel containing EVs-free media (DMEM supplemented with 2% FBS, with or without 200 µg/mL geneticin) at 37 °C in 5% CO_2_/95% humidified air. Spent media (40%) were collected every four days in two weeks; the collected media were replaced with fresh media every collection. Isolation of EVs from the collected media involved differential centrifugation to remove dead cells and debris. The media were subjected to the following conditions: (1) 500× *g*, 5 min; (2) 2000× *g*, 10 min; (3) 10,000× *g*, 30 min, at 4 °C. The cleaned media were then subjected to ultracentrifugation at 100,000× *g* for 2 h at 4 °C. After centrifugation, the EVs pellet was either resuspended in 1× PBS or lysed using radioimmunoprecipitation assay (RIPA) buffer with protease and phosphatase inhibitors and stored in −80 °C. EVs collected from un-transfected HEK293T cells are designated as HEK293T E, and from NB60-HEK293T cells as NB60 E, from here on.

### 2.6. Characterization of Collected EVs

EVs resuspended in distilled water were used for the Nanoparticle Tracking Analysis (NTA) to estimate the number and size of the particles as well as the zeta potential of the collected EVs. Another batch of EVs were used for Fluorescent NTA where the EVs were incubated with anti-CD9 (Cat # FAB1880G) and CD63-AlexaFluor488 (Cat # IC5048G, BioTechne, Minneapolis, MN, USA) antibody for 2 h at 4 °C with shaking, as reported earlier [39]. Lysates from EVs were prepared for Western blotting. The RIPA-lysed EVs were used to prepare protein samples. These protein samples were probed for both EVs (Alix, TSG101, CD81, CD9) and non-EVs (Calnexin, NF-κB, Vimentin) markers. Protein lysates from HEK293T cells were used as non-EVs control [40].

### 2.7. Proteomics Analysis for EV Protein Cargo

Based on protein quantification results, 30 µg proteins from NB60 E were isolated on S-trap micro column (Protifi, K02-micro). The isolated proteins were alkylated and digested on column based on manufacturer’s instructions. Then all the samples (triplicates) were vacuumed dried and submitted to FSU Translational Science Laboratory. The samples were analyzed on the Thermo Q Exactive HF, as previously described. Briefly, resulting raw files were searched with Proteome Discoverer 2.4 using SequestHT, Mascot, and Amanda as search engines. Scaffold (version 5.0) was used to validate the protein and peptide identity. Peptide identity was accepted if Scaffold Local false discovery rate (FDR) algorithm demonstrated a probability greater than 99.0%. Likewise, protein identity was accepted if the probability level was greater than 99.0% and contained a minimum of two recognized peptides. Gene Ontology (GO) annotation was carried out by g:Profiler.

### 2.8. Expression of Trop2 in Different Cell Lines

Cell suspension containing 1.5 × 10^6^ cells of either LTLT-Ca or MDA-MB-468 were seeded into a 100 mm dish in their respective media and grown overnight. Cells were then collected and lysed using RIPA buffer with protease and phosphatase inhibitors. Protein samples were prepared and were subjected to Western blotting to probe Trop2 using Trop2 antibody (Cat # 47866, Cell Signaling technology, Inc., Danvers, MA, USA).

### 2.9. Trop2 Knockdown by NB60 E and Effect on Cell Viability

Cell suspension containing 1.5 × 10^6^ cells of LTLT-Ca or MDA-MB-468 cells were seeded into a 100 mm dish in their respective media and allowed to adhere overnight. The cells were then treated with 1 × 10^11^ particles/mL of either HEK293T E or NB60 E for 48 h. In this study, NB60 E was evaluated at a single concentration (1 × 10^11^ particles/mL), which was selected based on the IC_50_ value obtained from the MTT assay. After EVs treatment, the cells were lysed to obtain protein lysates and were then subjected to Western blotting for Trop2 expression checking. To compare the effect on the cell viability of the EVs (HEK293T E or NB60 E) in LTLT-Ca cells, cell viability assay was performed. Cell suspension containing 7 × 10^3^ LTLT-Ca cells were seeded into a 96-well plate and were allowed to adhere overnight. Different concentrations of EVs (HEK293T E and NB60 E) (0, 1.0 × 10^9^, 2.0 × 10^9^, 3.8 × 10^9^, 6.9 × 10^9^, 1.37 × 10^10^, 2.75 × 10^10^, 5.5 × 10^10^, and 1.1 × 10^11^ particles/mL) were prepared and treated into the cells for 48 h. After EVs treatment, MTT (3-(4,5-dimethylthiazol-2-yl)-2,5-diphenyltetrazolium bromide) assay was performed according to the provided protocol; briefly, 5 mg/mL of MTT solution was added into the plate, incubated for 3 h at 37 °C in 5% CO_2_/95% humidified air. The absorbance was measured at 570 nm using a Tecan Infinite 200 PRO M Plex multimode microplate reader (Tecan, Männedorf, Switzerland). The data were plotted using GraphPad Prism version 8.0 for Windows (San Diego, CA, USA).

### 2.10. Trop2 Knockdown by Sacituzumab (Sac) Versus NB60 E

LTLT-Ca cell suspensions containing 1.5 × 10^6^ cells were seeded into 100 mm tissue culture dishes and incubated overnight to allow cell attachment. The following day, cells were treated with Sacituzumab (Cat. #HY-P999045, MedChemExpress, Princeton, NJ, USA) at concentrations of 50, 100, or 200 nM, or with NB60 E at particle concentrations of 1 × 10^10^ or 1 × 10^11^ particles/mL. After 48 h of treatment, cells were harvested, lysed, and protein extracts were prepared for Western blot analysis to assess Trop2 expression levels. Cells that were not treated with either Sac or NB60 E served as the control.

### 2.11. Effect of NB60 E on Downstream Trop2 Signaling Pathways

LTLT-Ca cell suspension containing 1.5 × 10^6^ cells were seeded into a 100 mm dish and allowed to adhere onto the plate overnight. The cells were then treated with 5 × 10^10^ particles/mL of either the HEK293T E or NB60 E for 48 h. After EVs treatment, the cells were lysed, and protein lysates were collected. Several protein markers related to cell proliferation/cell cycle (total and phosphorylated ERK 1/2, cyclin D1, and FoxM1), AKT/PARP pathway/apoptosis (total and phosphorylated AKT, cleaved PARP, caspase 3), IL6/Stat pathway (total and phosphorylated Stat3, IL-6), and epithelial–mesenchymal transition (EMT)-related signaling pathway (vimentin, TGF-β, total and phosphorylated NF-κB) were probed using Western blot. Cells that were not treated with EVs served as the control. The data were plotted using GraphPad Prism version 8.0 for Windows (San Diego, CA, USA).

### 2.12. Effect of NB60 E on Cytokine Production

Jurkat cell suspension containing 1.5 × 10^5^ cells were seeded into a 12-well suspension plate. Activation of the cells was performed by treating them with 50 ng/mL of phorbol 12-myristate 13-acetate (PMA) and 1 µM of ionomycin overnight at 37 °C in 5% CO_2_/95% humidified air. The media was replaced with a fresh media before treatment with 1 × 10^11^ particles/mL of each respective EVs, HEK293T E, or NB60 E. Cell culture supernatant was collected at 24 h and 48 h post-EVs treatment. Particulates such as dead cells and debris were removed from the collected cell culture supernatant by centrifugation. The supernatants were tested for cytokine levels using Human IL-2/IL-6/TNF-α ELISA kit (Invitrogen, Life Technologies, Carlsbad, CA, USA) according to the manufacturer’s protocol. Cells that were activated but no EVs treatment served as the control. The data were plotted using GraphPad Prism version 8.0 for Windows (San Diego, CA, USA).

### 2.13. Western Blot Assay

Protein lysates collected from the cells or EVs were quantified using bicinchoninic acid (BCA) assay. Equal amount of protein (25 or 30 µg) was mixed with Laemmli buffer and β-mercaptoethanol, heated at 95 °C for 5 min, and chilled on ice for 3 min. The reduced proteins were then fractionated by sodium dodecyl sulfate polyacrylamide gel electrophoresis (SDS-PAGE) (Any kD™ Mini-PROTEAN^®^ TGX Stain-Free™ Protein Gels, Cat # 4568123, Bio-Rad, Richmond, CA, USA) and transferred to nitrocellulose membrane (Cat # 1620097, Bio-Rad, Richmond, CA, USA). Then, the membranes were blocked using 5% bovine serum albumin (BSA) in tris-buffered saline with tween 20 (TBS-T) for 1 h and incubated with respective primary antibody at 4 °C overnight. After primary antibody incubation, the membranes were washed three times using with 1× TBS-T and then were treated with the secondary antibody, either anti-rabbit or anti-mouse IgG, HRP-linked antibodies for 1 h at room temperature. Proteins were visualized using Pierce ECL Western Blotting Substrate (Cat # 32209, Sigma-Aldrich, St. Louis, MO, USA) and imaging of proteins was performed using the ChemiDoc XRS+ System (Bio-Rad, Hercules, CA, USA). Protein levels were determined by quantifying the chemiluminescent intensities using Image Lab 6.0 (Bio-Rad, Hercules, CA, USA) and normalizing against the corresponding β-actin band intensities. The data were plotted using GraphPad Prism version 8.0 for Windows (San Diego, CA, USA).

### 2.14. In Vitro Angiogenesis Assay

Studies have shown that Trop2 expression is linked to the promotion of angiogenesis. To confirm this, in vitro angiogenesis assay was performed. Briefly, NB60 E-enriched supernatant from LTLT-Ca cells were prepared. To do this, a suspension with 1 × 10^5^ cells were seeded into each well of a 12-well plate. When the cells adhered to the plate, they were treated with the respective EVs (HEK293T E or NB60 E) at concentration of 5 × 10^10^ particles/mL for 48 h. Supernatant was collected and centrifuged to remove cell debris. A total of 3 × 10^4^ human umbilical vein endothelial cells (HUVEC) resuspended in 200 μL of corresponding supernatant (HEK293T E or NB60 E supernatant), complete media without treatment (negative control), and complete media with 50 ng/mL epidermal growth factor (EGF) (positive control) were plated into a 96-well plate previously coated with 100 μL Matrigel (Corning, Bedford, MA, USA). The effect on the tube formation of the NB60 E was evaluated after 8-h incubation and images were captured using OLYMPUS IX73 microscope, Tokyo, Japan. Effects on tube formation were evaluated by counting the number of nodes, junctions, branches, and branching lengths using NIH ImageJ software (version 1.54).

### 2.15. In Vivo Imaging

To evaluate the tumor-targeting efficiency of NB60 E toward Trop2-expressing tumors, the EVs were labeled with the near-infrared fluorescent dye Cy7-DSPE (BP-41494, BroadPharm, San Diego, CA, USA). The labeling was performed by incubating the EVs with Cy7-DSPE at a ratio of 100:1 (EVs/dye) overnight. Following incubation, the labeled EVs were purified by ultracentrifugation at 100,000× *g* for 90 min at 4 °C. The resulting EVs pellet was then resuspended in phosphate-buffered saline (PBS).

A total of 100 µg of the labeled EVs in 100 µL PBS was administered intraperitoneally (i.p.) into three mice. In vivo fluorescence imaging was performed using the Analytik Jena UVP iBox imaging system (Analytik Jena, Upland, CA, USA) to monitor the biodistribution and tumor uptake of the labeled EVs. Images were captured at multiple time points 0, 2, 4, 6, and 24 h post-injection. The accumulation of EVs in the tumor region was assessed by quantifying the fluorescence intensity using the instrument’s built-in analysis software. Control group were mice treated with Cy7-DSPE alone.

### 2.16. In Vivo Knockdown of Trop2 by NB60 E

To establish tumor xenografts, 4 × 10^6^ LTLT-Ca cells suspended in 50 μL of culture medium were mixed with 50 μL of Matrigel and subcutaneously injected into the right flank of BALB/c athymic nude mice. Estradiol valerate was successfully incorporated into a cyclodextrin-based formulation. Specifically, 6 mg of estradiol valerate was dissolved in 10% ethanol, followed by the addition of 10% Kolliphor^®^ EL. In parallel, 60 mg of hydroxypropyl-β-cyclodextrin was dissolved in phosphate-buffered saline (PBS) and vortexed for 1–2 min. The aqueous cyclodextrin solution was then combined with the organic phase containing estradiol valerate and Kolliphor^®^ EL, and the resulting mixture was vortexed for an additional 1–2 min. The formulation was administered via both subcutaneous injection and oral route (formulation was mixed with drinking water) at a dose of 35 mg/kg to promote tumor growth. Once the tumors reached a volume of approximately 500–600 mm^3^, NB60 E (1 × 10^11^ particles per animal) was delivered via i.p. injection. This dose was selected based on IC_50_ values obtained in vitro and is consistent with previously reported exosome dosing ranges in vivo [39,40]. The intraperitoneal route was chosen to facilitate systemic distribution and enable reproducible administration. Tumors were subsequently excised and processed for protein extraction to perform Western blot analysis. Mice in the control group (n = 3) did not receive NB60 E treatment.

### 2.17. Statistical Analysis

To determine statistical significance, the values of each treatment group were compared to the respective controls either by Student’s *t*-test for comparisons between two variables or by One-Way ANOVA with Dunnett’s post hoc test for multiple comparisons using GraphPad Prism version 8.0 (San Diego, CA, USA) and *p* < 0.05 was considered significant.

## 3. Results

### 3.1. Establishment of NB60-Expressing Cells

Successful transfection of pNB60 (Figure 1A) into HEK293T cells was verified by Western blotting, wherein a prominent band size (25 kDa) corresponding to NB60 was observed in pNB60-transfected cells but not in un-transfected cells (Figure 1C). To guarantee NB60-expressing HEK293T cells, geneticin selection for 30 days was performed, after which NB60 expression was further confirmed with Western blot (Figure 1D). NB60-expressing cell lines were maintained in selection media (DMEM supplemented with 2% FBS, with 200 μg/mL geneticin) in the proceeding experiments.

### 3.2. Metabolic Analysis of NB60-HEK293T Cell Growth in the PBS-Vertical Wheel Bioreactor

**Figure 2:**
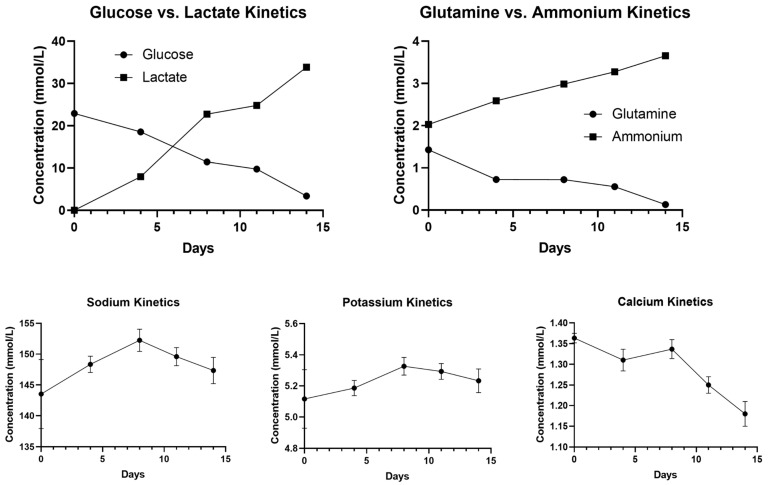
Metabolic activity of pNB60-transfected HEK293T cell in PBS-vertical wheel bioreactor. Metabolites were measured from media sampled from the bioreactor for 14 days. The levels of primary metabolic compounds by products such as glucose, lactate, glutamine, ammonium, as well as electrolytes including sodium, potassium, and calcium, were measured using BioProfile Flex2 analyzer.

To maximize the EVs yield from NB60-HEK293T cells for subsequent in vitro and in vivo studies, we cultured the cells for 14 days in a stirred-tank bioreactor and sampled the medium daily to track their metabolic profile and pinpoint optimal harvest times. Glucose declined steadily from 22.92 mmol/L on day 0 to 18.83, 11.45, 6.34, and finally 3.40 mmol/L on days 4, 8, 12, and 14, respectively. Lactate rose inversely, climbing from 0 to 7.96, 22.73, 29.99, and 33.84 mmol/L over the same period. By day 8, the lactate-to-glucose molar ratio approached 2.0, the upper limit for predominantly aerobic metabolism, signaling that a metabolic shift was imminent. Glutamine mirrored the glucose trend, falling from 1.43 mmol/L at inoculation to 0.73, 0.72, 0.40, and 0.13 mmol/L, while ammonium accumulated from 2.03 to 2.59, 2.99, 3.54, and 3.65 mmol/L. The marked depletion of both glucose and glutamine by days 4 and 8 prompted us to do a 40% medium exchange every four days in subsequent bioreactor runs. Electrolyte monitoring revealed parallel drops in Na^+^, K^+^, and Ca^2+^ beginning on day 8, confirming the onset of declining metabolic activity (Figure 2). Media collected before this decline produced the highest EVs titers and was therefore selected for downstream isolation.

### 3.3. Characterization of Isolated EVs

**Figure 3:**
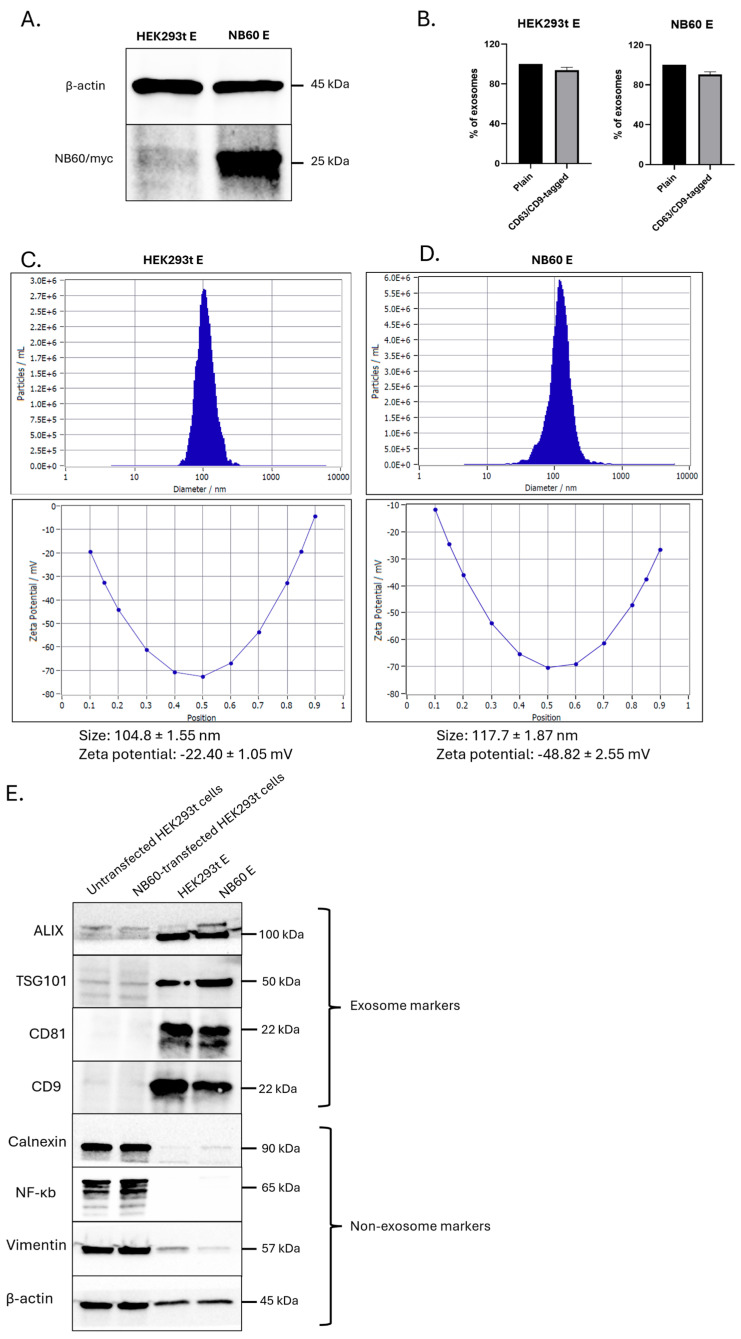
Characterization of isolated EVs. (**A**) Proteins were extracted from isolated EVs, HEK293T E, and NB60 E. Expression of NB60 in the NB60 E was confirmed by Western blotting. (**B**) Percentage of fluorescent EVs tagged with CD9/CD63 fluorescent antibodies. (**C**) NTA results characterizing HEK293T E. (**D**) NTA results characterizing NB60 E. (**E**) Western blot results of un-transfected HEK293T, NB60-transfected HEK293T cells HEK293T E and NB60 E, respectively.

EVs were isolated from both un-transfected HEK293T cells (HEK293T E) and pNB60-transfected cells (NB60 E) and then rigorously characterized. First, immunoblotting with an anti-myc antibody (the NB60 sequence was tagged with myc) revealed a distinct approximately 25 kDa band exclusively in the NB60 E samples, confirming successful NB60 sequence display on the vesicle (Figure 3A). Fluorescent NTA, performed after dual labeling with Alexa Fluor 488-conjugated anti-CD9 and anti-CD63 antibodies, showed that ~94% of all particles carried these canonical EVs markers, verifying the identity of the isolates (Figure 3B). Physicochemical analysis indicated that HEK293T E vesicles had a mean diameter of 104.8 ± 1.55 nm, a zeta potential of −22.40 ± 1.05 mV, and a concentration of 2.7 × 10^11^ particles/mL (Figure 3C). NB60 E vesicles were slightly larger (117.7 ± 1.87 nm), more negatively charged (−48.82 ± 2.55 mV), and present at 2.3 × 10^11^ particles/mL (Figure 3D). In both preparations, only a minor fraction fell outside the expected 40–160 nm exosomal range. Finally, Western blotting detected the EVs-specific proteins Alix, TSG101, CD81, and CD9 in the vesicle fractions but not in the parent cells, while calnexin, NF-κB, and β-actin, markers of cellular contamination were absent (Figure 3E). Together, these data confirm that the isolates are pure, well-defined EVs and that NB60 is efficiently incorporated without markedly altering vesicle size or yield.

### 3.4. NB60 E Cargo Analysis by Proteomics

**Figure 4:**
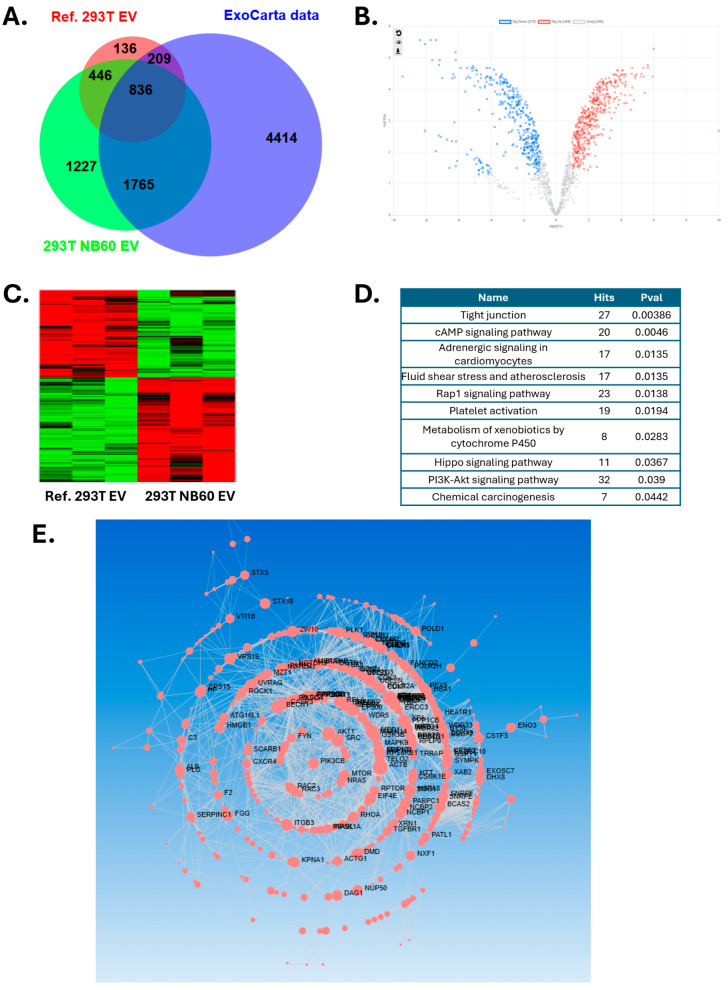
Proteomic analysis of HEK293T NB60 E. (**A**) Venn diagram of proteins identified in NB60 E (green), 293T EV from reference (red) and EV proteins from ExoCarta database (blue). (**B**) Volcano plot of DEPs comparing with reference. (**C**) Heatmap of DEPs. (**D**) KEGG pathway analysis of DEPs. (**E**) Interaction network analysis of NB60 E unique proteins.

NB60 E proteins were purified and subjected to proteomic analysis. The identified proteins were compared with proteomic data from ultracentrifugation (UC)-purified normal HEK293T extracellular vesicles (EVs) from a previously published reference [41], as well as with EV protein data from the ExoCarta database (Figure 4A). A total of 836 proteins were found to be common across all three datasets, including well-known EV marker proteins such as CD81, TSG101, and ITGB1. In addition, 1227 proteins were uniquely identified in the NB60 E dataset and were not present in either the reference dataset or the ExoCarta database. Indicating that NB60 E carry a significantly altered protein cargo, likely reflecting the engineered or pathological state of the parent cells. These unique proteins could represent novel biomarkers or EV-mediated signaling mediators not previously associated with HEK293t EV populations. Although there are differences in the cargoes between the two EVs, the presence of the canonical EV markers in NB60 E imply that these vesicles are real EVs and the altered cargo is not due to contamination but represents a true functional divergence of the bioengineered EVs. When comparing the NB60 E proteome with that of HEK293T EVs, 1282 overlapping proteins were identified, among which 819 were differentially expressed proteins (DEPs), 370 were downregulated, and 449 were upregulated (Figure 4B,C). These DEPs reflect a rewiring of the EV proteome which could potentially impact the interaction of the vesicles with recipient cells. Two important biological processes that might be influenced by these changes are cell proliferation and survival. Pathway enrichment analysis using the Kyoto Encyclopedia of Genes and Genomes (KEGG) database revealed that these DEPs may be involved in the regulation of the cyclic AMP (cAMP) and PI3K/Akt signaling pathways, both of which may potentially interact with the Trop2 signaling pathway (Figure 4D). The PI3k/Akt pathway is a well-established axis in cancer wherein its activation promotes cell survival, migration, and resistance to stress. cAMP, on the other hand, is linked to hormone signaling and transcriptional regulation and interacts with PI3K/Akt signaling, leading cells to either grow or die. Since these pathways may crosstalk with the Trop2 signaling cascade, which leads to tumor progression and epithelial cell proliferation, such interaction suggests that NB60 E could influence oncogenic signaling via coordinated regulation of these pathways. Furthermore, analysis of the protein–protein interaction (PPI) network built from all NB60-unique proteins (comprising the 1227 mentioned above plus 1765 additional proteins; see Figure 4A) highlighted Phosphatidylinositol 4,5-bisphosphate 3-kinase catalytic subunit beta (PIK3CB) as one of the central nodes in the network (Figure 4E), suggesting a key regulatory role. Taken together, we hypothesize that the altered proteome of the NB60 E suggests they might have the capacity to alter recipient cells by delivering regulatory proteins involved in survival and growth of cells.

### 3.5. Trop2 Expression Was Knocked Down by the NB60 E and Decreased the Viability of LTLT-Ca Cells

**Figure 5:**
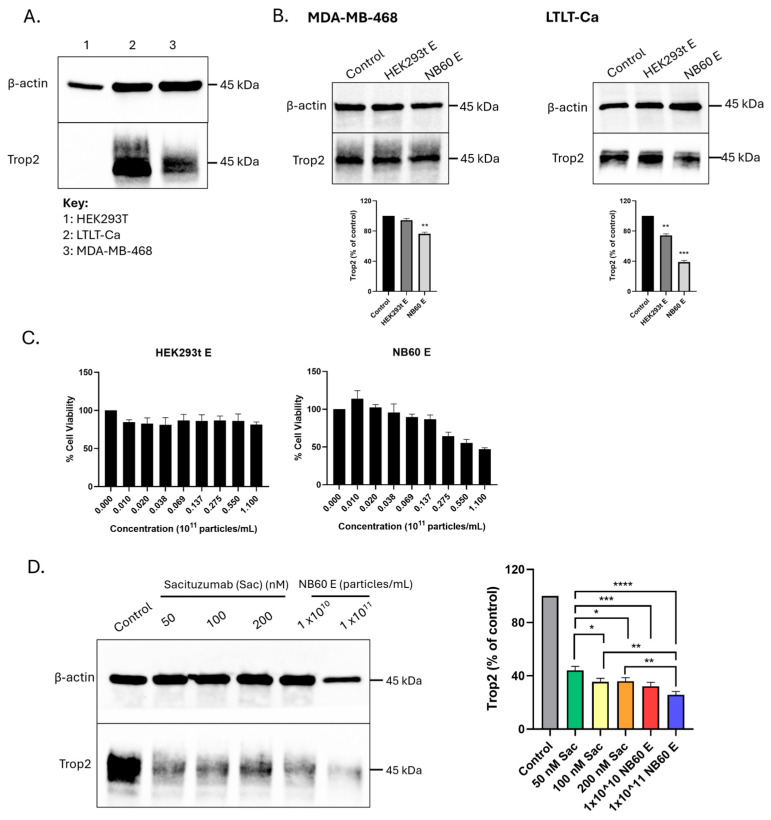
Knockdown of Trop2 expression by NB60 E and cell viability against LTLT-Ca cells. (**A**) Cell lines expressing Trop2, namely, MDA-MB-468 and letrozole-resistant MCF-7 (LTLT-Ca), were checked for Trop2 expression by Western blot. Expression was compared relative to the control cells HEK293t, which do not express Trop2. (**B**) Respective cell lines were treated with either HEK293T E or NB60 E for 48 h. After EVs treatment, protein lysates were collected and ran for Western blot. Trop2 knockdown was compared to the control, the cells not treated with the respective EVs. (**C**) LTLT-Ca cells were seeded onto a 96-well plate and were treated for 48 h with varying concentrations of the respective EVs. After treatment of cells with the indicated concentrations of EVs, MTT assay was performed to determine the viability of the cells. (**D**) LTLT-Ca cells were seeded into 100 mm tissue culture dishes and were treated for 48 h with either Sacituzumab (Sac) or NB60 E. After treatment, the cells were collected, and protein lysates were prepared for Western blot. Trop2 expression was probed to compare knockdown of the said antibodies in LTLT-Ca cells. Cells that were not treated with either Sac or NB60 E served as the control group. (*, *p* < 0.05; **, *p* < 0.01; ***, *p* < 0.001; ****, *p* < 0.0001).

Breast cancer cell lines, letrozole-resistant MCF-7 (LTLT-Ca) and MDA-MB-468, cells, expressing Trop2 were used to determine whether the NB60 E was capable of blocking Trop2 expression. Both cell lines tested showed high expression of Trop2, more specifically, LTLT-ca cells (Figure 5A). After NB60 E treatment, Trop2 expression was effectively blocked in by 23.5 ± 1.5% in MDA-MB-468, and 61.5 ± 1.5% in LTLT-Ca, relative to the control (Figure 5B). Of the two cell lines, LTLT-Ca showed the most significant Trop2 knockdown, thus, these cells were used for further experiments. Trop2 expression was effectively inhibited by the NB60 E and not by the HEK293T E. To determine if blocking Trop2 affects its downstream functions, we checked on the effect of NB60 E treatment on the viability of LTLT-Ca cells. Results showed treatment of the cells with 1 × 10^11^ particles/mL of HEK293T E did not significantly affect the cells (Figure 5C); however, NB60 E killed the cells by 52.8 ± 0.9%, relative to the controls (cells that were not treated with the EVs) (Figure 5C).

### 3.6. Knockdown of Trop2 by NB60 E Was as Efficient as Sacituzumab

To confirm the efficacy of NB60 E in knocking down Trop2 expression in LTLT-Ca cells, the cells were treated with either NB60 E or a commercial Trop2 antibody, Sacituzumab (Sac). NB60 E treatment with 1 × 10^10^ or 1 × 10^11^ particles/mL of EVs resulted in 67.7 ± 1.7 and 74.1 ± 1.4% reduction in Trop2 expression, respectively, relative to the control. The significant reduction was comparable to the cells treated after treatment with varying concentrations of Sac. Cells treated with 50, 100, and 200 nM of Sac markedly reduced the expression of Trop2 by 55.9 ± 1.7, 64.4 ± 1.4, and 63.9 ± 1.3%, respectively (Figure 5D). The results implicate the knockdown capability of the NB60 E as much as the commercially available Trop2 antibody, Sacituzumab.

### 3.7. NB60 E Impeded the Expression of Proteins Downstream of Trop2 Involved in Signaling Pathways

**Figure 6:**
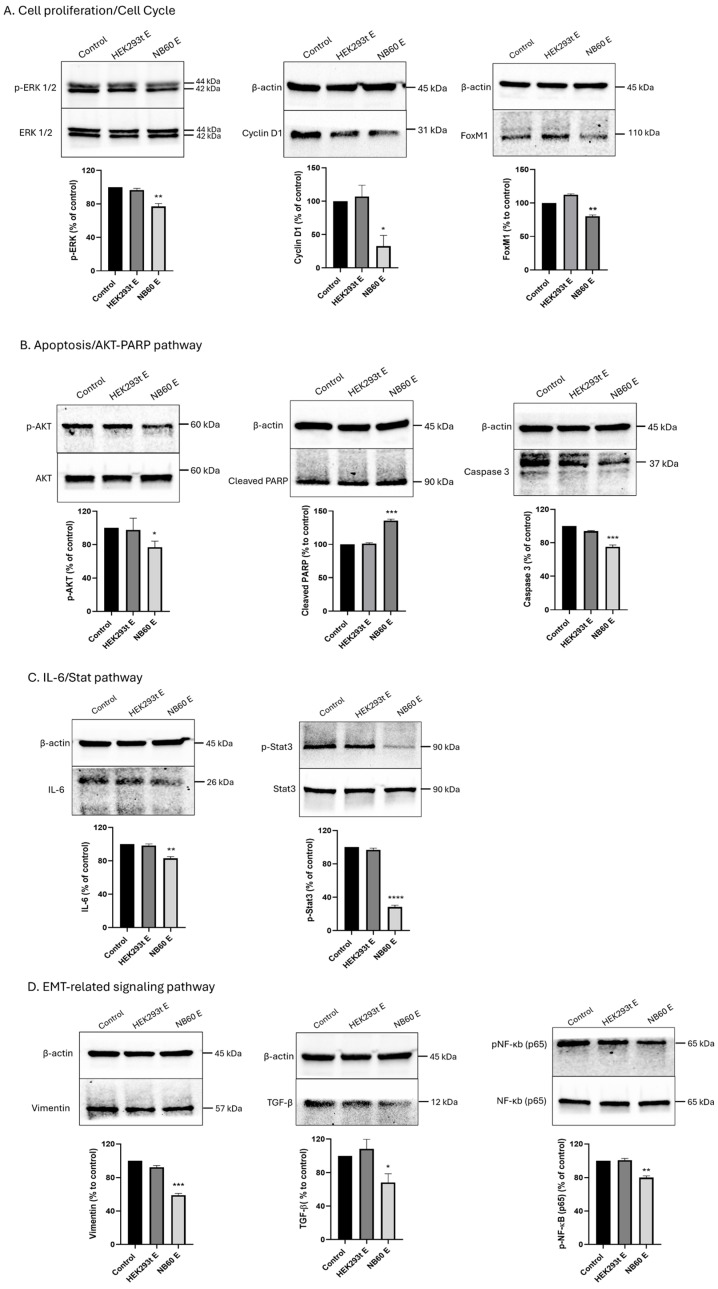
Effect of NB60 E on downstream Trop2 signaling pathways. LTLT-Ca cells were seeded onto a 100 mm plate and were treated for 48 h with 5 × 10 ^10^ particles/ ml of respective EVs (HEK293T E or NB60 E). After treatment of cells with EVs, the cells were collected, and protein lysates were prepared for Western blot. Different proteins related to cell signaling were probed, specifically, (**A**) cell proliferation/cell cycle, (**B**) apoptosis/AKT-PARP pathway, (**C**) IL-6/Stat pathway, and (**D**) EMT-related signaling pathway. Cells that were not treated with EVs served as the control. (*, *p* < 0.05; **, *p* < 0.01; ***, *p* < 0.001; ****, *p* < 0.0001 vs. control).

As was demonstrated from the cell viability assay, knockdown of Trop2 impedes proliferation of the LTLT-Ca cells. To identify which specific pathways downstream of Trop2 that are directly involved in cancer progression are affected after NB60 E treatment, we probed for different proteins involved in cell signaling. ERK 1/2 was not affected by NB60 E treatment; however, a decrease in p-ERK 1/2 by 30 ± 4% was observed. Cyclin D1 and Fox M1 level were significantly decreased by 67 ± 11% and 21.4 ± 17% after NB60 E treatment (Figure 6A). Results also showed a decrease in p-AKT and caspase level by 23.4 ± 4.3% and 24.8 ± 1.5%, respectively, and increase by 34.2 ± 1.3% of cleaved PARP (Figure 6B). The effect of NB60 E was also determined in IL-6/Stat3 signaling pathway; results revealed a decrease in IL-6 and phosphorylated Stat3 level by 17.1 ± 1.4% and 71.8 ± 1.6%, respectively, but no effect on Stat3 (Figure 6C). We also investigated the effect of NB60 E on some EMT markers. Results revealed a decrease in vimentin, TGF-β, and phosphorylated NF-κB level by 40.8 ± 1.4, 31.8 ± 7.5, and 20.1 ± 1.5%, respectively (Figure 6D) (*, *p* < 0.05; **, *p* < 0.01; ***, *p* < 0.001).

### 3.8. NB60 E Affected Cytokine Levels in Jurkat T Cells

**Figure 7:**
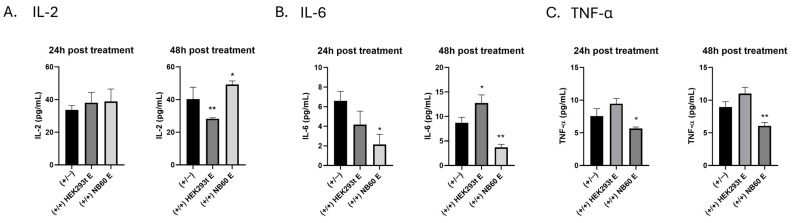
Effect of NB60 E on cytokine production in Jurkat T cells. Cells were seeded onto a 12-well suspension plate and activated with 50 ng/mL of PMA and 1 µM of ionomycin overnight. The activated cells were then treated with respective EVs (HEK293T E or NB60 E) and supernatant was collected at two time points (24 and 48 h) post-EVs treatment. The levels of (**A**) IL-2, (**B**) IL-6, and (**C**) TNF-α were quantified using ELISA. (+/−) are cells that were activated with PMA and ionomycin but no EVs treatment, and served as the control (*, *p* < 0.05; **, *p* < 0.01 vs. control).

Here, we determined the levels of three relevant cytokines involved in tumor progression: (a) IL-2, (b) IL-6, and (c) TNF-α. Our results showed that EVs treatment increased IL-2 level 24 h post-treatment, NB60 E-treated cells showed 38.8 ± 3.8 pg/mL, while HEK293T E-treated cells had 38.1 ± 3.2 pg/mL, compared to the control, activated/no EVs (+/−) with 33.7 ± 1.3 pg/mL. However, at 48 h post-EVs treatment, a significant increase by 22.1 ± 2.7% with 49.2 ± 1.1 pg/mL was observed in NB60 E-treated cells compared to the controls, activated/no EVs treatment (+/−) with 40.28 ± 3.6 pg/mL and HEK293T E with 28.28 ± 0.3 pg/mL (Figure 7A). Moreover, a significant reduction in IL-6 levels was observed in Jurkat T cells 24 and 48 h post-NB60 E treatment compared to the control (+/−). Specifically, at 24 h post-treatment, NB60 E-treated cells showed a decrease by 68.1 ± 7.3% with 2.1 ± 0.6 pg/mL, while the HEK293T E-treated cells had 4.2 ± 0.8 pg/mL, which is 43.1 ± 7.5% lower than the control (+/−). At 48 h post-EVs treatment, IL-6 level increased in HEK293T-E treated cells with 12.8 ± 1 pg/mL, 46.8 ± 6.5% higher than the control (+/−). However, NB60 E-treated cells IL-6 level decreased by 57.5 ± 1.5% with 3.7 ± 0.4 pg/mL (Figure 7B). A decrease was also observed in the TNF-α similar to IL-6 after 24 and 48 h post-NB60 E treatment. At 24 h, the amount of TNF-α in the NB60 E-treated cells was 5.7 ± 0.5 pg/mL, which is 25 ± 0.7% lower than the control with 7.6 ± 1.6 pg/mL, while the HEK293T E had 9.5 ± 1.2 pg/mL of the cytokine, which is 22.1 ± 2.3% higher than the control. After 48 h post-treatment, NB60 E-treated cells had 6.1 ± 1.2 pg/mL of the cytokine, which is 32.1 ± 0.3% lower than the control with 8.9 ± 0.8 pg/mL; however, HEK293T E-treated cells increased by 24.2 ± 1.4% with 1.8 pg/mL (Figure 7C) (*, *p* < 0.05; **, *p* < 0.01).

### 3.9. NB60 E Blocked Angiogenesis in HUVECs

**Figure 8:**
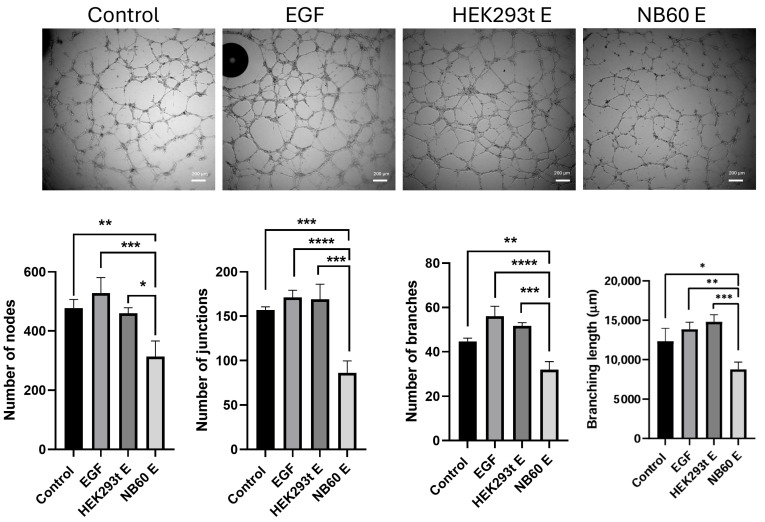
Effect of NB60 E on angiogenesis in HUVECs. LTLT-Ca cells were seeded onto a 12-well plate and treated with respective EVs (HEK293T E and NB60 E) for 48 h. The supernatant was then collected and centrifuged to remove cell debris. HUVECs resuspended in respective EVs-enriched supernatant (HEK293T E or NB60 E supernatant) were then plated into 96-well plate, previously coated with 100 μL of Matrigel. The untreated HUVECs served as the negative control while the positive control group was treated with 50 ng/mL of epidermal growth factor (EGF). Images were taken after 8-h incubation using NI OLYMPUS IX73 microscope, and tubular formation was analyzed using NIH ImageJ software (version 1.54) (*, *p* < 0.05; **, *p* < 0.01; ***, *p* < 0.001, ****, *p* < 0.0001).

We determined if the NB60 E treatment affects the formation of new vessels. Results revealed significant suppression of tubular formation of HUVECs treated with NB60 E. The evaluation of tubular formation was assessed by counting the number of nodes, junctions, and branches and the branching length. Noticeably, NB60 E-treated HUVECs showed fewer nodes, junctions, and branches with 313 ± 30.6, 86.2 ± 7.7, and 32 ± 2.1, respectively, compared to the negative control, EGF-, and HEK293T E-treated cells (Figure 8). NB60 E-treated cells also showed a shorter branching length of 877.3 μM than the other groups.

### 3.10. NB60 E Localized in the Tumor

**Figure 9:**
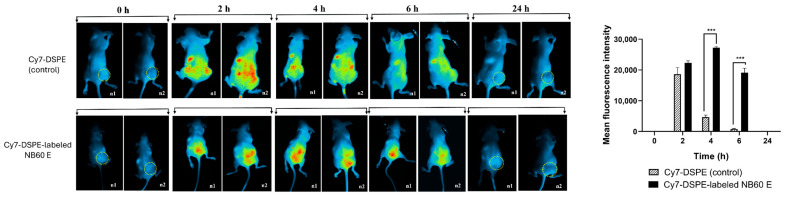
In vivo tracking of NB60 E in the tumor. BALB/c athymic nude mice were subcutaneously injected with LTLT-Ca cells on their right flank region. When tumor growth becomes visible (550 mm^3^) (encircled), Cy7-DSPE-labeled EVs (100 μg in 100 μL PBS, NB60 E—1 × 10^11^ particles/animal) were intraperitoneally injected into the mice. Imaging of the live mice was performed at different time points (0, 2, 4, 6, 24 h). Fluorescence intensity was measured using the built-in software of the instrument, Analytik Jena UVP iBox (Analytik Jena, Upland, CA, USA). Control group are mice treated with Cy7-DSPE alone (***, *p* < 0.001).

NB60 E were engineered using a nanobody sequence specific to Trop2, enabling targeted interaction with Trop2-overexpressing tumor cells. To assess the tumor-targeting capability and biodistribution of NB60 E, the EVs were labeled with the near-infrared fluorescent dye Cy7-DSPE and administered to tumor-bearing mice. In vivo imaging revealed a progressive accumulation of fluorescence in the tumor region beginning 1 h post-injection, suggesting successful uptake of the EVs. The fluorescence intensity increased significantly by 2 h and reached its peak at approximately 4 h post-administration. A gradual decline in signal was observed by 6 h, and by 24 h post-injection, the signal had nearly disappeared, indicating systemic clearance of the EVs (Figure 9). These results confirm the specificity of NB60 E for Trop2-expressing tumors and support the hypothesis that these EVs can serve as effective targeted delivery vehicles. Furthermore, the reduced presence of Cy7-DSPE-labeled EVs at 24 h suggests efficient clearance from the body, thereby potentially reducing off-target effects in surrounding tissues.

### 3.11. NB60 E Inhibited Trop2 Expression in Mice

**Figure 10:**
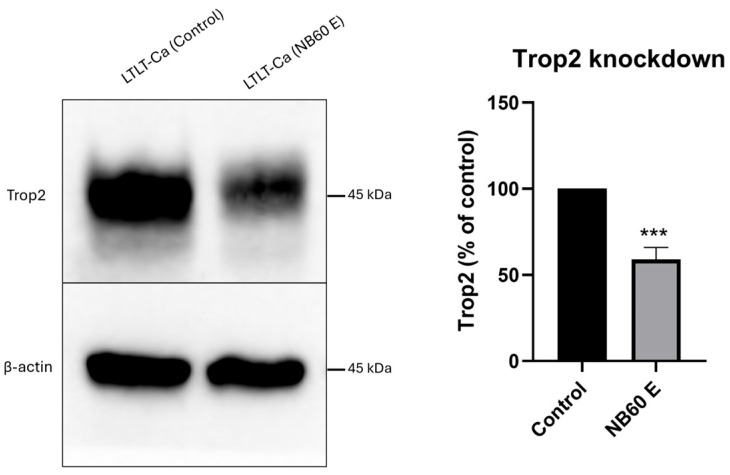
In vivo knockdown of Trop2 by NB60 E. BALB/c athymic nude mice were subcutaneously injected with LTLT-Ca cells on their right flank region. When tumor growth becomes visible (550 mm^3^), NB60 E (1 × 10^11^ particles/animal) were injected intraperitoneally. After 24 h of EVs treatment, the tumor was excised, and protein lysates were collected for Western blotting. Tumors collected from mice not treated with NB60 E serve as the control (***, *p* < 0.001 vs. control). Legend: LTLT-Ca (Control)—tumor without EVs treatment, LTLT-Ca (NB60 E)—tumor treated with NB60 E.

NB60 E is effectively bound to the tumor region of mice, as shown in Figure 9. Investigation on its ability to block the expression of Trop2 in the tumor area was conducted, and the result showed a successful knockdown of Trop2 by 41 ± 4% relative to the control tumor (Figure 10). The result confirms our hypothesis that NB60 E explicitly targets Trop2-enriched tumors and can inhibit Trop2 expression (***, *p* < 0.001).

## 4. Discussion

Breast cancer (BC) is the most common type of cancer and remains a leading cause of cancer-related mortality among women. Incidence rates were observed to be confined to localized-stage and hormone receptor-positive disease. According to the most recent breast cancer statistics, one in eight women or 13% will be diagnosed with invasive BC, and one in 43, or 2% will die from the disease in the United States [42]. Approximately 80% of breast cancers are estrogen receptor (ER)-positive, thus making endocrine therapy the standard care of treatment along with surgery in the majority of breast cancer cases [43]. Advances in BC therapy, especially chemotherapy and/or endocrine therapy with the use of hormones for those with hormone receptor-positive BC, helped managed BC patients. Over the past three decades, improvements in screening, hormonal blockade, and combination chemotherapy have steadily lowered mortality; nonetheless, acquired resistance and metastatic relapse continue to undermine long-term survival. It is therefore important to investigate how drug resistance occurs and to discover alternative chemotherapeutic agents or combinations of targeted therapies to improve survival rates of BC patients.

One of the most compelling molecular candidates for targeted intervention is trophoblast cell surface antigen 2 (Trop2). Microarray and immunohistochemical surveys reveal Trop2 overexpression in approximately 83% of BC specimens relative to normal breast tissue, and functional studies across multiple laboratories have demonstrated that Trop2 drives cancer-cell proliferation in vitro and tumor expansion in vivo [5,13,44]. Moreover, Trop2 expression correlates to increased tumor growth regardless of histotype (carcinoma and sarcoma) and species (human and murine), suggesting Trop2 as a conserved driver of tumor growth [13]. The clinical success of Sacituzumab govitecan, an ADC consisting of humanized anti-Trop2 monoclonal antibody (hRS7), conjugated with active metabolite of irinotecan (SN-38 provided the first FDA-approved example of Trop2-targeted therapy for metastatic triple-negative BC (mTNBC)).

Despite this advancement, traditional monoclonal antibodies (mAbs) suffer from two intrinsic shortcomings: their large size (~150 kDa) limits diffusion through the dense extracellular matrices of solid tumors, and their production in mammalian systems is costly and time-consuming [45]. Camelid-derived nanobodies (VHHs) avoid both issues. Weighing roughly 15 kDa, nanobodies penetrate tumor parenchyma more efficiently, clear rapidly from systemic circulation, and can be manufactured inexpensively in microbial hosts [46,47]. A Trop2-focused nanobody–drug conjugate has already shown potent antitumor activity in pancreatic-cancer xenografts [48], and iterative bio-panning has yielded additional nanobodies, including the sequence on which our NB60 clone is based, that suppress proliferation and migration of Trop2-positive colon-cancer cells [49]. These observations prompted us to explore whether combining nanobody specificity with a biologically compatible delivery system could produce a next-generation Trop2-directed therapeutic. We therefore engineered extracellular vesicles—endogenous, 40–160 nm vesicles noted for their excellent biocompatibility, natural tropism, and ability to traverse biological barriers—to express a Trop2-specific nanobody sequence (NB60) on EVs.

It is possible that soluble NB60 nanobody could mediate similar effects; however, they face significant challenges. Liposomes, solid lipid nanoparticles, and lipid–polymer hybrids often exhibit limited stability during storage, low drug-loading efficiency, rapid clearance by the reticuloendothelial system, and potential immunogenicity or off-target effects [50,51]. Furthermore, batch-to-batch variability, difficulties in large-scale production, and high manufacturing costs continue to limit their practical applications [52,53]. EVs-based delivery confers several distinct advantages: (a) EVs possess intrinsic homing capabilities due to their lipid composition and tetraspanin expression, which may promote preferential uptake by tumors or immune cells, enhancing bioavailability at the tumor site; (b) EVs represent a modular platform; while NB60 E functions without additional cargo in this study, future applications could integrate co-delivery of siRNA, micro RNA, drugs, or immunomodulators, thus offering a versatile and expandable therapeutic architecture.

The pDisplay vector, which contains an Ig κ-chain leader sequence for secretion and a platelet-derived growth-factor receptor (PDGFR) transmembrane anchor, was used to fuse the NB60 coding region in frame, ensuring that the nanobody sequence would be displayed on the plasma membrane of transfected HEK293T cells and packaged onto secreted vesicles [54]. Western blots confirmed nanobody expression on both cells and their EVs (Figure 1C and Figure 3A). Although EVs have been co-opted for various drug delivery and immunotherapy applications [55], the present work is, to our knowledge, the first to harness bioengineered EVs specifically for Trop2 targeting, introducing a wholly new vesicle-based modality for this antigen.

In this study, we employed a genetic engineering strategy that utilized the pDisplay vector, which fuses the nanobody NB60 to the PDGFR transmembrane domain rather than the more commonly used LAMP-2B. This choice was deliberate for several reasons. First, PDGFR anchoring ensures stable membrane integration and external orientation of the nanobody sequence without interfering with endogenous EVs’ protein-sorting machinery, which has been reported with LAMP-2B fusion constructs. Second, the pDisplay system includes a κ-chain leader sequence that promotes efficient membrane trafficking and surface presentation in both the transfected cells and their released EVs. Finally, genetic engineering at the donor cell level allows for continuous, scalable, and uniform production of functionalized EVs, which is more reproducible than post-isolation chemical conjugation strategies that often suffer from low yield, poor orientation control, or structural instability of ligands.

Our process-development studies showed that the productivity and quality of EVs expressing NB60 are tightly coupled to the metabolic state of the producer cells. When NB60-HEK293T cultures were transferred from flasks to a 500 mL vertical wheel bioreactor, glucose concentration fell from 22.9 mmol/L on day 0 to 11.5 mmol/L by day 8, while lactate climbed reciprocally to 22.7 mmol/L. At that point the lactate-to-glucose molar ratio approached 2.0, the upper limit generally considered compatible with predominantly aerobic metabolism; ratios > 2 signal a switch to anaerobic glycolysis and impending acid stress. A comparable trend was observed for glutamine, which decreased from 1.43 to 0.72 mmol/L, accompanied by an increase in its catabolic byproduct, ammonium, from 2.0 to 3.0 mmol L. Electrolytes such as Na^+^, K^+^ and Ca^2+^ also declined from day 8 onward, consistent with the onset of declining cellular activity (Figure 2). The data informed two process adjustments that proved critical for downstream work. First, we instituted a 40% medium exchange every four days, beginning on day 4, to replenish carbon and nitrogen sources while diluting lactate and ammonium. Second, we harvested conditioned medium for EVs isolation before day 10, when aerobic metabolism still prevailed. Batches collected after the metabolic shift yielded ~25% fewer vesicles and showed broader size distributions, a finding that aligns with reports that lactate accumulation and pH drift compromise vesicle biogenesis and membrane integrity in HEK293 cultures. Taken together, the metabolic study enabled a rational fed-batch strategy that maximized NB60 E recovery while preserving vesicle quality. Because producer-cell metabolism varies with cell line, vessel geometry, and medium formulation, the same workflow-tracking glucose, lactate, glutamine, ammonium, and key electrolytes to pinpoint the aerobic-to-anaerobic inflection-should be broadly applicable to the scalable production of other ligand-decorated EVs. Notably, NB60 E displayed a more negative zeta potential (−48.82 ± 2.55 mV) than control EVs (−22.40 ± 1.05 mV). The observed increase in the negative zeta potential of NB60 E may be attributed to changes in surface charge distribution due to the presence of engineered membrane proteins. Similar findings were reported by Sawada et al., who demonstrated that RSV peptide engineering on dendritic cell-derived EVs significantly altered zeta potential in a charge-dependent manner, confirming that surface modifications can modulate EVs electrokinetic properties without compromising vesicle morphology or quality [56]. Western blot analysis of purified NB60 E revealed a distinct 25 kDa band corresponding to the size of the Trop2 nanobody.

Our experimental plan was three-pronged. First, we evaluated whether NB60 E could bind Trop2 on letrozole-resistant LTLT-Ca breast-cancer cells and suppress antigen expression. Second, we examined downstream effects on canonical cancer hallmarks, proliferation, angiogenesis, epithelial–mesenchymal transition (EMT), and cytokine signaling in vitro. Third, we assessed in vivo biodistribution by labeling EVs with the near-infrared probe Cy7-DSPE. NB60 E localized to tumor tissue within one hour of intraperitoneal injection, reached maximal fluorescence at four hours, and cleared by twenty-four hours with minimal off-site retention [49], confirming efficient and selective delivery.

Mechanistic investigation revealed that NB60 E-mediated Trop2 blockade suppressed multiple oncogenic signaling pathways. Proteomic study comparing with control 293T EV from reference showed several significantly changed pathways, including tight junction, cAMP signaling, fluid shear stress, cytochrome P450, and PI3K-Akt signaling. We were using a vertical wheel bioreactor to harvest EVs from 293T-NB60 cells, which may change the EV protein profile on the tight junction and fluid shear force. Adding geneticin to maintain NB60 expression will change the cell metabolism and promote expression of P450 for drug metabolism. Finally, these unique proteins expressed in the 293T-NB60 EV may have functions in the PI3K-Akt signaling pathways, which was further proved by our Western blotting results. We had demonstrated marked reductions in phosphorylated ERK1/2 and cyclin D1, key effectors of the MAPK pathway, consistent with previous findings in cervical [16], gall-bladder [14], non-small-cell lung [57], and triple-negative breast-cancer systems [58]. Concomitant declines in colony formation, invasion, and three-dimensional Matrigel invasion corroborated the functional significance of these molecular changes. LTLT-Ca cells, derived from ER^+^ MCF-7 but rendered letrozole-resistant after prolonged aromatase-inhibitor exposure, are characterized by MAPK hyperactivation, enhanced stem-cell frequency, EMT, and hormone independence [36,59,60]. NB60 E treatment substantially decreased EMT-associated proteins (vimentin, TGF-β, phosphorylated NF-κB), suggesting partial reversal of endocrine resistance and induction of apoptotic susceptibility.

The observed downregulation of Trop2 protein following treatment with NB60 E is likely due to ligand-induced receptor internalization and degradation, a phenomenon that has been described in the context of antibody or nanobody targeting of surface proteins. Such observation can be confirmed in Figure 4D, where we compared the binding ability of a commercial Trop2 antibody (Sacituzumab) with the NB60 E. The bioengineered EVs exhibited binding to Trop2, knocking it down as much as Sacituzumab. Sacituzumab (50 nM) was included as a reference comparator to benchmark the activity of NB60 E. Given that Sacituzumab is an antibody and NB60 E are exosomes, the concentrations cannot be directly correlated; therefore, this comparison should be interpreted as qualitative rather than dose equivalent. Trop2 is a membrane-bound glycoprotein known to undergo clathrin-mediated endocytosis upon ligand or antibody engagement [10]. It is plausible that NB60 E binding to Trop2 mimics this natural ligand interaction, thereby triggering internalization and subsequent lysosomal degradation of the receptor. This concept is supported by previous studies in which Trop2-targeting antibodies or ADCs led to receptor downregulation and impaired signaling [21]. While we did not directly visualize Trop2 internalization in this study, the significant reduction in surface and total protein levels across multiple cell lines suggests receptor modulation rather than transcriptional suppression. Future studies involving confocal microscopy and lysosome inhibitors will further clarify the precise internalization–degradation pathway induced by NB60 E.

Because Trop2 intersects inflammatory signaling, we next measured key cytokines after activating Jurkat T cells. NB60 E raised IL-2 levels, a cytokine known to expand and activate natural killer cells, thereby enhancing their cytotoxicity against BC cells [61,62]. Conversely, NB60 E lowered IL-6 and TNF-α, two cytokines that promote EMT, aromatase expression, and aggressive clinical behavior [63,64,65]. Specifically, we note that Jurkat T cells do not express Trop2; therefore, the observed cytokine modulation is likely mediated through indirect mechanisms rather than Trop2 targeting. One possible explanation is the activation of extracellular vesicle-associated pattern recognition receptors (PRRs). Because the bioengineered NB60 E display non-self-proteins, their interaction with PRRs may engage surface receptors on Jurkat cells and initiate downstream signaling cascades. Our Western blot data demonstrating decreased NF-κB protein levels support this hypothesis, as reduced NF-κB activity is consistent with suppression of pro-inflammatory cytokines such as IL-6 and TNF-α. At the same time, the observed increase in IL-2 secretion may be explained by preferential activation of the Ca^2+^/NFAT signaling pathway, which is known to selectively promote IL-2 transcription. Taken together, these findings suggest that NB60 E exert an immunomodulatory effect through a shift in transcriptional control from NF-κB-driven pro-inflammatory signaling toward NFAT-mediated IL-2 production. While these results provide an initial mechanistic rationale, we acknowledge that additional studies will be required to delineate the precise molecular pathways involved. Compared with un-transfected HEK293T-EVs controls, NB60 EVs therefore reshape the cytokine profile from broadly pro-inflammatory to one dominated by IL-2 with suppressed IL-6 and TNF-α outputs, underscoring Trop2’s role as the principal mediator of this immunomodulatory shift. In parallel, NB60 E impaired endothelial tube formation in HUVECs, consistent with the literature showing that Trop2 fosters angiogenesis through ERK activation in NSCLC [57] and glioma [66].

At a deeper signaling level, Trop2 has been linked to calcium-dependent activation of MAPK and up-regulation of cyclin D1 [67,68]. Trop2 also activates CREB1, Jun, NF-κB, and STAT transcription factors [10], implicating inflammation to oncogenesis [69,70,71,72,73,74,75]. NB60 E attenuated phosphorylated NF-κB and STAT3, in tandem with the aforementioned drop in IL-6 and TNF-α, indicating that the NB60 E affects both proliferative and inflammatory functions of cancer cells.

These data collectively show that NB60 E binds Trop2 with high specificity, down-modulates the antigen, and diminishes multiple downstream pathways that sustain tumor growth, metastasis, and immune evasion. Importantly, the vesicular platform does so without the size-imposed diffusion barriers or manufacturing burdens characteristic of full-length antibodies. Given the ubiquity of Trop2 overexpression across solid tumors, the versatility of exosomal surface engineering, and the modularity of nanobody payloads, the strategy outlined here could be rapidly adapted to other Trop2-driven malignancies or to multi-targeted EVs designs that incorporate additional therapeutic cargos.

While our in vitro results show a robust antitumor response to NB60 E, in vivo efficacy and safety validation are essential for confirming therapeutic translatability. Preliminary in vivo data from our biodistribution and tumor Trop2 knockdown experiments show that NB60 E selectively accumulates in Trop2-positive tumors and downregulates Trop2 by 41%, validating its targeting ability. However, we acknowledge the need to expand these studies using systemic delivery models, orthotopic tumor models, and longer-term monitoring of tumor growth inhibition, survival benefit, and toxicity. Additionally, dose-escalation and biodistribution studies, especially evaluating liver, kidney, and immune system effects, are necessary to assess safety and immunogenicity. Such work is underway in our lab and will form the basis for future preclinical evaluations.

Inclusion of free NB60 as a control is essential to decouple the effects of nanobody vs. exosomal delivery. This is a limitation of the current study. While Hu et al. [38] demonstrated that NB60 alone does not inhibit proliferation, our data show the exosomal display of NB60 results in Trop2 knockdown and downstream signaling suppression, suggesting a possible mechanism dependent on multivalent presentation and enhanced cellular uptake. Unlike soluble nanobodies that can only target antigens monovalently (one nanobody binds to one epitope), exosome-displayed nanobodies are presented in a multivalent configuration on the exosomal membrane. Thus, each exosome can carry dozens or hundreds of the nanobody molecules on the surface. This multivalent display effectively enhances avidity, which is the overall binding strength resulting from multiple simultaneous interactions. Even if individual nanobody–Trop2 interactions are of moderate affinity, the combined effect of many concurrent bindings produces a much stronger, more stable interaction with the target cell surface. The avidity of exosome-displayed NB60 to Trop2 is much stronger than soluble nanobodies; this high-avidity interaction facilitates stronger and longer-lasting binding of exosomes to Trop2-positive cells, thereby promoting receptor clustering, which, in turn, triggers endocytosis and downstream receptor degradation. We hypothesize that the observed Trop2 knockdown by the exosome-displayed NB60 was due to its high avidity to Trop2, which increased internalization and turnover of receptor, and such interactions are observed to be less efficient with monovalent, soluble nanobody formats. We acknowledge that NB60 in soluble form may be biologically inert in vitro, but exosomal surface engineering transforms its activity profile. We are currently developing comparative studies including free NB60 treatment to directly test this hypothesis in vitro and in vivo.

Our results strongly suggest that bioengineered extracellular vesicles are effective in targeting cancer cells. However, concerns about off-target effects and immunogenicity currently limit their clinical use. Since EVs can be easily taken up by a wide range of cells through processes like endocytosis or membrane fusion, there is a risk that they may leak or deliver their cargo non-selectively outside of the intended target tissues or therapeutic areas. This could lead to unwanted changes in gene expression or cause cell death and inflammation. Additionally, engineered EVs may display non-self-proteins, which can be recognized as foreign by the host’s immune system, potentially resulting in inflammation or cytokine release syndrome (CRS). Therefore, a careful design process, the use of humanized nanobodies, and thorough in vivo validation are essential before these engineered EVs can be commercially utilized. While our current study demonstrates that NB60 E effectively modulates Trop2 expression and cytokine signaling in tumor models, further work is needed to fully characterize its therapeutic potential. Future studies will focus on long-term efficacy, pharmacokinetics, and safety profiling in animal models, including extended dosing regimens, biodistribution analyses, and comprehensive toxicity assessments. These investigations will provide critical insights into the translational applicability of NB60 E and help guide optimization for potential clinical development.

In summary, we report the first study to utilize Trop2-bioengineered EVs for targeted therapy in breast cancer. NB60 E selectively homes to Trop2-overexpressing, letrozole-resistant cells, suppresses MAPK and inflammatory signaling, inhibits proliferation and angiogenesis, and re-balances TME cytokines toward an antitumor phenotype. These findings position NB60 E as a promising biologic for overcoming endocrine resistance and warrant further pharmacokinetic, safety, and efficacy evaluation in advanced preclinical models.

## 5. Conclusions

The occurrence of Trop2 in most tumors and its involvement in several signaling pathways associated with tumorigenesis solidify its stature as a marker for target therapy. Developments of new anti-cancer-targeted therapies are underway, and many promising Trop2-centric interventions are currently being explored. To our knowledge, this is the first study that developed Trop2-targeted bioengineered EVs. The ability of the NB60 E to target cancer cells clearly demonstrates its potency as a new anti-cancer therapy. The clinical translational potential of these engineered EVs hinges on their ability to deliver therapeutic payloads safely and effectively, such as miRNAs or siRNAs, without causing cytotoxicity. Furthermore, it is important that the platform is consistently manufactured (reproducible and cost-effective) and can be administered to patients. Future studies include using these EVs to carry drug payload, not only specific to breast cancer but to most Trop2-expressing cancer types.

## Data Availability

The datasets generated during and/or analyzed during the current study are available from the corresponding authors upon request. The mass spectrometry proteomics data have been deposited to the ProteomeXchange Consortium via the PRIDE partner repository with the dataset identifier.
